# Loss of floral repressor function adapts rice to higher latitudes in Europe

**DOI:** 10.1093/jxb/erv004

**Published:** 2015-03-01

**Authors:** Jorge Gómez-Ariza, Francesca Galbiati, Daniela Goretti, Vittoria Brambilla, Roshi Shrestha, Andrea Pappolla, Brigitte Courtois, Fabio Fornara

**Affiliations:** ^1^University of Milan, Department of Biosciences, Via Celoria 26, 20133 Milan, Italy; ^2^University of Milan, Department of Agricultural and Environmental Sciences – Production, Landscape, Agroenergy, Via Celoria 2, 20133 Milan, Italy; ^3^CIRAD, UMR AGAP, Avenue Agropolis, 34398 Montpellier Cedex 5, France; ^4^ Current Address: Università Cattolica del Sacro Cuore, Via Emiliana Parmense 84, Piacenza, Italy

**Keywords:** Adaptation, *Ehd1*, *Hd1*, *Hd3a*, heading date, photoperiodic flowering, rice, *RFT1*.

## Abstract

The capacity to discriminate variations in day length allows plants to align flowering with the most favourable season of the year. This capacity has been altered by artificial selection when cultivated varieties became adapted to environments different from those of initial domestication. Rice flowering is promoted by short days when *HEADING DATE 1* (*Hd1*) and *EARLY HEADING DATE 1* (*Ehd1*) induce the expression of florigenic proteins encoded by *HEADING DATE 3a* (*Hd3a*) and *RICE FLOWERING LOCUS T 1* (*RFT1*). Repressors of flowering antagonize such induction under long days, maintaining vegetative growth and delaying flowering. To what extent artificial selection of long day repressor loci has contributed to expand rice cultivation to Europe is currently unclear. This study demonstrates that European varieties activate both *Hd3a* and *RFT1* expression regardless of day length and their induction is caused by loss-of-function mutations at major long day floral repressors. However, their contribution to flowering time control varies between locations. Pyramiding of mutations is frequently observed in European germplasm, but single mutations are sufficient to adapt rice to flower at higher latitudes. Expression of *Ehd1* is increased in varieties showing reduced or null *Hd1* expression under natural long days, as well as in single *hd1* mutants in isogenic backgrounds. These data indicate that loss of repressor genes has been a key strategy to expand rice cultivation to Europe, and that *Ehd1* is a central node integrating floral repressive signals.

## Highlight

Allelic variants of floral repressor genes have been artificially selected to reduce sensitivity to photoperiod of rice varieties cultivated in Europe, allowing cultivation of a tropical species at higher latitudes.

## Introduction

Photoperiod (or day length) and temperature are crucial cues that plants use to monitor diurnal and seasonal time (Amasino, 2010). Complex molecular networks have evolved in distinct plant lineages to translate environmental signals into molecular signals that drive reproductive development (Andrés and Coupland, 2012). Rice (*Oryza sativa*) is a facultative short day (SD) plant in which flowering is accelerated upon exposure to photoperiods falling below a critical threshold, and has been extensively used as model system to understand the genetic and molecular basis of flowering time (Brambilla and Fornara, 2013).

Besides its prominent role as a plant model system, amenable to genetic studies, rice is a staple food for large parts of the world population, particularly in Asian countries, where rice was originally domesticated and cultivated. Recent molecular data based on re-sequencing of several hundred accessions of cultivated and wild rice, indicate that the centre of rice domestication can be traced to the middle area of the Pearl River Basin, in southern China (Wei *et al.*, 2012; Huang *et al.*, 2012). From these tropical regions, rice cultivation has enormously expanded, reaching temperate areas in Asia, up to 55°N (Shrestha *et al.*, 2014). During the 15th century, rice cultivation became established in Europe and Italy is currently the major rice producer. As opposed to tropical Asia, cultivation of rice in temperate regions is constrained by low winter temperatures that restrict the cropping season to late spring and summer, when the photoperiod is long and not inductive. Artificial selection has allowed breeding of rice varieties adapted to higher latitudes, improving germinability and tolerance to low temperatures (Fujino *et al.*, 2008; Suh *et al.*, 2010), and reducing sensitivity to photoperiod (Xue *et al.*, 2008; Fujino *et al.*, 2013; Koo *et al.*, 2013).

The genetic architecture of flowering in rice depends on a regulatory network controlling expression of two proteins, encoded by *HEADING DATE 3a* (*Hd3a*) and *RICE FLOWERING LOCUS T 1* (*RFT1*), orthologs of *FLOWERING LOCUS T* (*FT*) of *Arabidopsis* (Kojima *et al.*, 2002; Turck *et al.*, 2008; Tsuji *et al.*, 2013). Upon perception of favourable photoperiods, *Hd3a* and *RFT1* are expressed in the vasculature of leaves and move to the shoot apical meristem to promote the switch from vegetative to reproductive development (Tamaki *et al.*, 2007; Komiya *et al.*, 2008; Komiya *et al.*, 2009). Expression of *Hd3a* and *RFT1* is induced by *HEADING DATE 1* (*Hd1*), a zinc-finger CCT domain transcription factor, and *EARLY HEADING DATE 1* (*Ehd1*), a B-type response regulator (Yano *et al.*, 2000; Izawa *et al.*, 2002; Doi *et al.*, 2004; Itoh *et al.*, 2010). Mutations in either *Hd1* or *Ehd1* cause delayed flowering under SD (Doi *et al.*, 2004). Conversely, when plants carrying *hd1* mutant alleles are grown under long day (LD) conditions flowering is induced earlier compared to the wild type, indicating that Hd1 can either promote or repress flowering, depending on the photoperiod (Yano *et al.*, 2000; Izawa *et al.*, 2002; Ishikawa *et al.*, 2011). The repressive activity of *Hd1* can be enhanced by *Hd6*, which encodes the *α* subunit of *CASEIN KINASE II* (*CKIIα*) (Takahashi *et al.*, 2001). Defective *Hd6* alleles are present in some *japonica* rice cultivars and reduce the capacity of Hd1 to repress flowering under LD (Takahashi *et al.*, 2001; Ogiso *et al.*, 2010; Ebana *et al.*, 2011; Shrestha *et al.*, 2014).

Several factors play prominent roles as regulators of flowering under LD, primarily acting upstream of *Ehd1*, to regulate its expression. Among them, *GRAIN YIELD, PLANT HEIGHT AND HEADING DATE 7* (*Ghd7*) and *Ghd8*/*DAYS TO HEADING 8* (*DTH8*) are potent repressors of *Ehd1* expression and strongly contribute to delay flowering (Xue *et al.*, 2008; Wei *et al.*, 2010; Yan *et al.*, 2011). *Ghd7* encodes a CCT domain protein that, when mutated, allows rapid flowering also under LD (Xue *et al.*, 2008). Phosphorylation of Ghd7 is necessary for its repressive activity and is mediated by *Hd16/EARLY FLOWERING 1* (*EL1*), encoding a casein kinase I (CKI) protein (Hori *et al.*, 2013). Loss-of-function alleles of *Hd16* have been isolated from Asian varieties capable of flowering early under LD conditions (Hori *et al.*, 2013; Kwon *et al.*, 2014). *Ghd8* encodes a homolog of the HEME ACTIVATOR PROTEIN 3 (HAP3) of *Arabidopsis*. Loss-of-function alleles at this locus also cause early flowering under LD, and have been artificially selected in several varieties to expand rice cultivation to northern areas (Wei *et al.*, 2010; Fujino *et al.*, 2013). *CONSTANS-LIKE 4* (*OsCOL4*) encodes a repressor of *Ehd1*, active under both SD and LD conditions, and no natural genetic variation at this locus has been reported yet (Lee *et al.*, 2010).

Cloning of *Hd2* showed that it encodes *PSEUDO RESPONSE REGULATOR 37* (*PRR37*), a repressor of *Hd3a* expression under LD (Koo *et al.*, 2013). Loss-of-function alleles at *PRR37* promote early flowering at northern latitudes by de-repressing *Ehd1* and the florigens (Gao *et al.*, 2014). These data indicate that the floral activation pathway is strongly repressed under long photoperiods and short day lengths activate flowering in part by relieving such repression. Mining of allelic variants of flowering time regulators in rice cultivars adapted to LD conditions has identified extensive natural genetic variation that has been selected by ancient farmers or breeders, as it conferred a selective advantage over the wild type alleles in temperate areas. The levels of genetic diversity among some flowering time genes in rice varieties that can flower in Europe have been partly explored (Naranjo *et al.*, 2014). However, it is not clear which level of genetic diversity exists among LD repressors in European varieties, or how it impacts on phenotypic variation across northern latitudes.

This study demonstrates that in several European varieties, expression of both *Hd3a* and *RFT1* is de-repressed regardless of photoperiod and causes a strong decrease of sensitivity to day length. Natural genetic variation at LD repressor loci largely explains such behaviour but the impact of loss-of-function alleles varies with latitude. These results indicate that fine tuning of photoperiod sensitivity has been a key strategy allowing expansion of rice cultivation to higher latitudes and provide useful data to geneticists willing to breed novel fast-cycling varieties for temperate environments.

## Materials and methods

### Plant material

Sixteen *temperate japonica* varieties cultivated in Italy (referred as the mini-panel in the text) were chosen for detailed genotyping and expression analyses. A set of 247 varieties extracted from the European Rice Germplasm Collection (http://eurigendb.cirad.fr), resulting from the pooling of the working collections of breeders from European public breeding institutions, was used for targeted association mapping (Supplementary Table S1). Two *Hd1* mutant lines in the Nipponbare background (NG6019 and NG0430) were obtained from the National Institute of Agrobiological Sciences of Japan (https://tos.nias.affrc.go.jp/; Miyao *et al.*, 2003). The insertions of *Tos17* in NG6019 (*hd1-1*) and NG0430 (*hd1-2*) were confirmed to be 577 and 1478bp after the transcription start site, respectively. Genotyping was performed using primers *Tos17*-For GAGAGCATCATCGGTTACATCTTCTC and *hd1-1*-Rev CACAGATTCCATCAGCAACAG, or *hd1-2*-Rev GCCAAATTCCAGAATCCTGA.

### Heading dates and PS index measurements

Heading dates were determined under SD (10L/14D) and LD (16L/8D) conditions in Conviron PGR15 growth chambers or in a walk-in growth chamber. Temperature and relative humidity were set at 28ºC/80% during the day and 24ºC/90% during the night, respectively. Seedlings used to measure heading dates under natural LD (NLD) were sown in a cold greenhouse under natural day length conditions on 5 April 2012 and 11 April 2013, and transplanted on 7 May 2012 and 14 May 2013, respectively, in an irrigated field at the University of Milan. About 20 to 30 seedlings per variety were transplanted and heading dates were scored from each individual.

Heading dates of the mini-panel under different day lengths were scored at emergence of the first panicle, just after opening of the leaf sheath. The Photoperiod Sensitivity index (PS) was calculated according to the formula: [(heading date under LD − heading date under SD) / heading date under LD]. For the 247 varieties listed in Supplementary Table S1, the phenotypic data of the CT95-37 RESGEN project ‘Constitution, description and dynamic management of rice genetic resources of European vocation’ supported by the European Community, DG VI, were used (Feyt *et al.*, 2001). The data were collected from experiments conducted between 1996 and 1998 in Arles (France), Alcala del Rio, in Andalusia (Spain), Thessaloniki (Greece) and Salvaterra de Magos in Estremadura (Portugal). Plants were grown under similar conditions in irrigated fields. The tested cultivars were arbitrarily divided into three groups in order to spread the work load over three years as described in Feyt *et al.* (2001). The experimental design was an augmented design with a common set of seven control varieties present all years in all trials. The control varieties (Ariete, Baldo, Cigalon, Loto, Koral, Senia and Thaibonnet) represented a broad range of flowering date variation from early to late. Dates of 50% flowering were recorded for all accessions and time to 50% flowering computed based on the trial sowing dates. Data were adjusted for the year effect. The Italy_2012 and Italy_2013 datasets were collected at the northernmost European location (Vercelli, Italy) in 2012 and 2013, respectively.

The final data used in association mapping analyses are presented in Supplementary Table S1.

### DNA extraction, PCR and sequencing

Young leaf pieces were pooled in 2ml tubes containing two clean steel balls. Tubes were rapidly frozen in liquid nitrogen and grinded using a tissue-lyser (Retsch). Genomic DNA from varieties of the mini-panel was extracted using a modified CTAB and chloroform:isoamyl alcohol method (Doyle and Doyle, 1987). The DNA pellet was resuspended in ddH_2_O and quantified using a NanoPhotometer Pearl (Implen). About 50–150ng of gDNA was used in each PCR reaction in a final volume of 13 µl. Primers were designed in order to sequence the coding regions (CDS) of *Hd1*, *PRR37*, *Ghd7*, *Ghd8* and *COL4*. The components were as follows: 1.3 µl of 10X PCR buffer, 2 µl of 5M Betaine or 1 µl of 30% DMSO, 0.2 µl of 10mM dNTPs, 0.25 µl of each primer (1 μg/μl, diluted 1:10), 0.2 µl Taq polymerase, and ddH_2_O to volume. For the amplification of fragments with high GC content, LA Taq and GC Buffers (Takara) were used following manufacturer’s instructions. Amplification reactions were run for 40 cycles. The PCR products were incubated with Exonuclease I (Thermo Scientific) and Fast Alkaline Phosphatase (Thermo Scientific) for 15min at 37°C. Enzymes were heat-deactivated for 15min at 85°C. Purified fragments were sequenced using specific primers.

### Candidate gene genotyping

Seedlings were grown in a greenhouse for two weeks. The aerial part was collected and grinded using steel balls in 2ml tubes after freezing with liquid nitrogen. Genomic DNA from all varieties was extracted using the Edwards method (Edwards *et al.*, 1991). A total of 17 different non-functional alleles in five different genes were genotyped using markers listed in Supplementary Table S2: six for *hd1*, three for *prr37*, three for *ghd7*, three for *ghd8* and two for *el1* (Supplementary Table S1). Primers for dCAPS design were obtained using dCAPS Finder 2.0 (http://helix.wustl.edu/dcaps/dcaps.html) allowing up to two mismatches. PCRs were conducted as described before, and digestions with restriction enzymes were carried out at 37°C overnight. The PCR products were separated by agarose gel electrophoresis, and visualized using a Fluorchem imaging system (Alpha Innotech) with Alpha Ease FC software. In association analyses, all loss-of-function alleles of a given gene were grouped into a single non-functional type (NF) (Supplementary Table S1).

### Quantification of gene expression and correlation between gene expression and heading dates

To quantify gene expression from developmental time courses, leaves were sampled at the indicated day, two hours after lights-on (ZT2). For diurnal time courses, leaves were sampled every three hours from three-week-old plants grown under under SD and six-week-old plants grown under LD. Leaf samples from plants grown under field conditions were collected during floral induction (from the middle of May until the end of July) every two weeks, two hours after dawn. Gene expression data from field-grown plants were collected in 2012 and 2013 with similar results. Data from 2012 are presented. Leaves were collected from at least three independent plants for each RNA preparation. Total RNA was isolated using either TRI reagent (Sigma-Aldrich) or the Spectrum Plant Total RNA Kit (Sigma-Aldrich) following manufacturer’s instructions. Total RNA was treated with DNase I using the TURBO DNase kit (Life Technologies), and precipitated with 0.1 volumes of sodium acetate and 2.5 volumes of 100% ethanol. The RNA pellet was washed with 75% ethanol and resuspended in ddH_2_O. RNA was quantified using a NanoPhotometer Pearl (Implen). 2 µg of total RNA were retrotranscribed using SuperScript II Reverse Transcriptase (Invitrogen) with oligo-dT and resuspended in a final volume of 140 μl of cDNA. Quantification of gene expression was carried out in a final volume of 10 µl using a Mastercycler Realplex^2^ (Eppendorf). qPCR reactions were carried out using 5 μl of 2X Maxima SYBR Green qPCR Master Mix (Thermo Scientific), 0.25 μl of each primer (1 μg/μl, diluted 1:10), 1.5 μl of ddH_2_O, and 3 μl of cDNA template. Primers used to quantify the mRNA levels of Ubiquitin, *Hd3a*, *RFT1*, *Hd1*, *Ehd1*, *Ghd7* and *PRR37* are listed in Supplementary Table S5. Correlations between heading date (days) and gene expression values of *Hd3a/Ubq* and *RFT1/Ubq* (log of relative gene expression) were determined by calculating the Pearson’s correlation coefficient (R^2^) using Microsoft Office Excel 2010.

### Association mapping

All accessions had been previously genotyped with SNPs spaced of at least 0.4Mb in the genome (Courtois *et al.*, 2012). A set of 85 SNPs polymorphic in the studied accession set, hereafter called ‘neutral SNP set’, was retained for further analysis. All analyses were conducted using Tassel v.4.0 (Bradbury *et al.*, 2007). A Principal Component Analysis was conducted on the neutral SNP set using a correlation matrix. Three axes together explaining 40% of the variation were retained. The matrix constituted of the loadings of the accessions on these three axes was used to control population structure. A kinship matrix was computed using the same dataset to capture more complex familial relationships. Several models were compared to test the significance of each SNP × trait association: a simple linear model without any control of population structure (GLM0), a linear model with control of structure (GLM1), a mixed model with control of kinship (MLM1) and a mixed model involving structure and kinship (MLM2). Only alleles with a frequency above 5% were used in the analyses. Taking into account the number of genes and polymorphisms within genes tested, a threshold of 0.005 was used to declare a test significant.

## Results

### Rice varieties adapted to Europe show reduced sensitivity to photoperiod

**Fig. 1.**
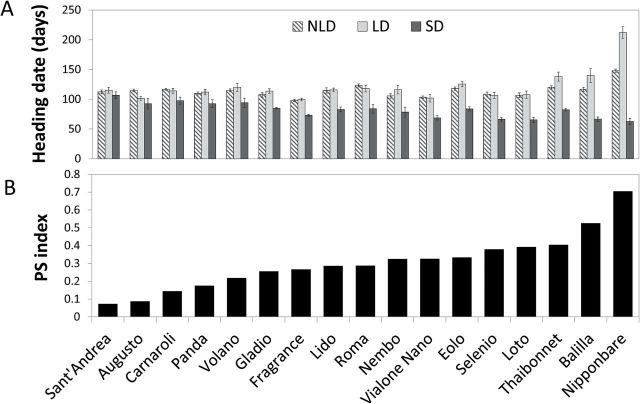
Heading dates and photoperiod sensitivity of 16 *temperate japonica* varieties cultivated in Italy and Nipponbare. (A) Days from germination to heading were scored under natural long days (NLD) in Milan, artificial long days (LD) and artificial short days (SD). (B) Photoperiod sensitivity index of the same varieties as in (A).

Artificial selection has expanded rice cultivation from tropical Asia to higher latitudes in Europe, partly by influencing the capacity of plants to flower under long days. To address the molecular mechanisms involved, a working panel composed of 16 *temperate japonica* varieties cultivated in Italy (mini-panel) was selected to be representative of the diversity of heading dates observed under natural field conditions (Fig. 1). The mini-panel and Nipponbare were grown under constant LD (16 hours light) and SD (10 hours light) in growth chambers, and under natural long day conditions (NLD) in Milan (~45°N). All varieties flowered earlier under SD conditions compared to LD conditions, and the response to distinct photoperiods showed large variation (Fig. 1A). Most varieties flowered at similar times under NLD and LD, indicating that photoperiodic flowering was similarly promoted both under constant and varying day lengths. Flowering of Thaibonnet, Balilla and Nipponbare was delayed under LD compared to NLD, indicating that these varieties could discriminate between different long photoperiods and that continuous LD of 16h caused stronger floral repression. Based on heading dates under LD and SD, we calculated a photoperiod sensitivity index (PS). All varieties tested showed reduced PS compared to Nipponbare (Fig. 1B), and some varieties, including Augusto and Sant’Andrea, were almost completely insensitive and flowered at the same time regardless of day length (Fig. 1A). These data indicate that flowering of mini-panel varieties can be promoted by SD but not repressed under LD and NLD compared to Nipponbare. Their capacity to discriminate between different day lengths is therefore impaired.

### Accessions belonging to the mini-panel carry non-functional alleles of long day floral repressor genes

Mutations in floral repressors have been associated to northern expansion of rice cultivation (Xue *et al.*, 2008; Fujino *et al.*, 2013; Hori *et al.*, 2013; Koo *et al.*, 2013; Kwon *et al.*, 2014; Shrestha *et al.*, 2014). The type and frequency of loss-of-function mutations was assessed at seven loci, encoding genes repressing flowering most effectively under LD and including *Hd1*, *PRR37*, *Ghd7*, *Ghd8*, *OsCOL4*, *Hd16/EL1* and *Hd6*/*CKIIα* (Yano *et al.*, 2000; Takahashi *et al.*, 2001; Xue *et al.*, 2008; Lee *et al.*, 2010; Yan *et al.*, 2011; Hori *et al.*, 2013; Koo *et al.*, 2013). The DNA sequence of all repressors was determined, including the CDS and some introns. However, the upstream regulatory regions were not sequenced, as polymorphisms in the promoters would be difficult to associate to loss-of-function alleles. Sequence data were compared to Nipponbare that bears functional alleles at all repressor loci with the exception of *Hd6* that bears a premature stop codon, resulting in a truncated non-functional protein (Takahashi *et al.*, 2001). Several allelic variants were identified (Table 1, Supplementary Fig. S1). Ten out of 16 varieties carry at least one loss-of-function allele at *Hd1*, *Ghd7* or *Ghd8*. Seven different alleles at the *Hd1* locus were detected. The 2 bp-deletion observed in Fragrance, Eolo, Thaibonnet, Gladio, Panda and Augusto (Hd1-V, Hd1-VI), and the 43bp-deletion observed in Lido and Selenio (Hd1-II) were previously isolated from the cultivars Kasalath and HS66 respectively, and were associated to loss-of-function alleles (Yano *et al.*, 2000). Balilla bears the same *Hd1* allele of Ginbouzu which was suggested to encode a stronger LD repressor allele than Nipponbare (Yano *et al.*, 2000). Four *PRR37* alleles were detected but none of them could be clearly associated to a loss-of-function variant (Supplementary Fig. S1). Four *Ghd7* alleles were identified, one of which showed a deletion of the locus and was previously described as *Ghd7-0* in early flowering rice varieties (Xue *et al.*, 2008) (Supplementary Fig. S1). The SNP at position 618 converts tyrosine into a Stop codon (*Ghd7-I*, Supplementary Fig. S1) and is reported here for the first time. The mutation is located in the CCT motif that is a critical functional domain for this class of transcription factors. The *Ghd8* locus was represented by four different alleles (Supplementary Fig. S1), two of which (*Ghd8-II* and *III*) were described as loss-of-function and associated to loss of photoperiod sensitivity (Wei *et al.*, 2010). Three genotypes were detected sequencing the CDS of *COL4* (Lee *et al.*, 2010), none of which was likely to alter the function of the gene. Genotyping of *Hd16* for two loss-of-function mutations previously identified in Japanese varieties (Type 2 and Type 3) (Kwon *et al.*, 2014) showed that no such variants were present within the mini-panel. Mutations in *Hd16* seem to have arisen locally and are not widely spread (Kwon *et al.*, 2014). Similarly, no mutations in *Hd6* were identified and all varieties carried a functional allele identical to that of Kasalath (Takahashi *et al.*, 2001).

**Table 1.** Genotypes of repressor genes in Italian cultivars comprised in the mini-panel

Variety	*Hd1* ^a^	*PRR37*	*Ghd7*	*Ghd8*	*COL4*	*Hd6*	*EL1^*b*^*
Sant’Andrea	III	I	Nip	II	Nip	Kas	wt
Augusto	VI	Nip	0	II	Nip	Kas	wt
Carnaroli	VII	I	Nip	Nip	Nip	Kas	wt
Panda	VI	II	0	I	I	Kas	wt
Volano	IV	I	Nip	Nip	Nip	Kas	wt
Gladio	VI	II	Nip	II	II	Kas	wt
Fragrance	VI	III	II	III	II	Kas	wt
Lido	II	I	Nip	Nip	Nip	Kas	wt
Roma	IV	I	Nip	Nip	Nip	Kas	wt
Nembo	VII	I	Nip	Nip	Nip	Kas	wt
Vialone Nano	VII	I	Nip	Nip	Nip	Kas	wt
Eolo	V	II	I	Nip	II	Kas	wt
Selenio	II	I	Nip	Nip	Nip	Kas	wt
Loto	IV	I	Nip	Nip	Nip	Kas	wt
Thaibonnet	V	II	Nip	Nip	II	Kas	wt
Balilla	I	I	Nip	II	I	Kas	wt
Nipponbare	Nip	Nip	Nip	Nip	Nip	Nip	wt

^a^ Shaded boxes indicate loss-of-function alleles. Roman numbers and 0 indicate allelic variants according to Supplementary Fig. S1. Nip and Kas indicate the alleles of Nipponbare and Kasalath, respectively.

^b^ The absence of type 2 and type 3 mutations in *EL1* is indicated as wt.

### Expression of *Hd3a* and *RFT1* is not repressed under long or short days in Italian varieties

Induction of *Hd3a* and *RFT1* is correlated to heading dates under controlled SD and NLD (Takahashi *et al.*, 2009; Naranjo *et al.*, 2014; Ogiso-Tanaka *et al.*, 2013). However, their seasonal expression dynamics have not been extensively determined for varieties bearing non-functional allelic variants of LD floral repressors, nor it is clear how accumulation of such mutations influences the flowering behaviour of varieties adapted to northern latitudes.

**Fig. 2.**
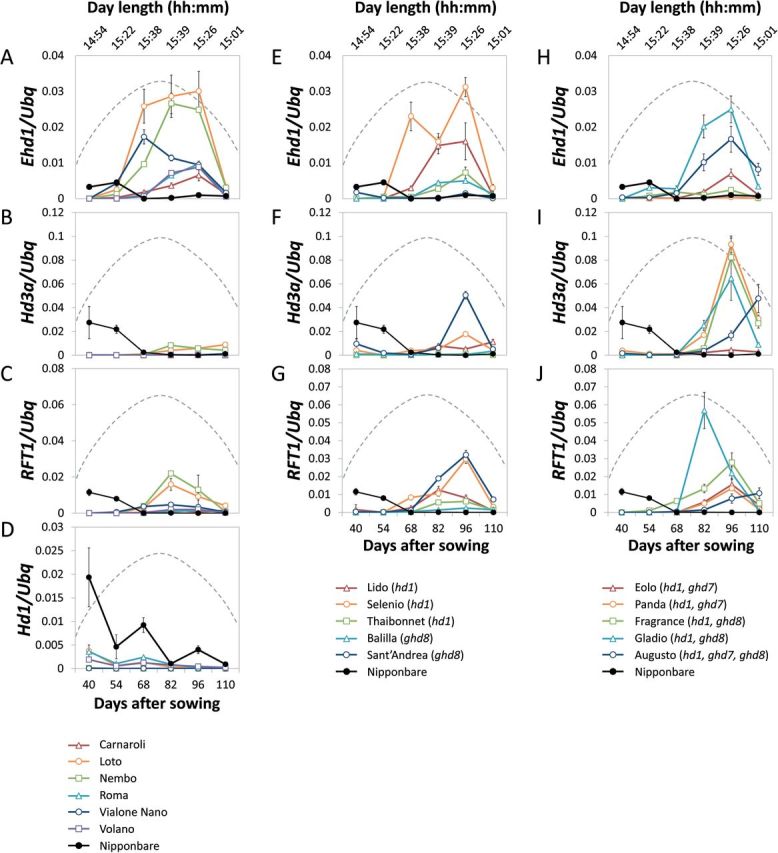
Seasonal patterns of *Hd3a* and *RFT1* expression in *temperate japonica* varieties cultivated in Italy and Nipponbare under natural field conditions at high latitudes (45°N). Expression dynamics of *Ehd1* (A, E, H), *Hd3a* (B, F, I), *RFT1* (C, G, J) and *Hd1* (D) in varieties bearing functional alleles of LD repressors (left), a single non-functional allele (center) or multiple combinations of LD floral repressors. The quantification of gene expression from Nipponbare was included as control and represented in all graphs to facilitate comparisons (black lines). All samples were collected 2h after dawn and values were normalized using *Ubiquitin* (*Ubq*) expression. The sampling period ranged from 15 May (40 DAS) to 24 September (110 DAS) during 2012. Biological replicates from 2013 gave similar results. Seasonal variation of day length is indicated with a dashed line and day length at sampling is indicated on top of the graphs. The longest day length during the sampling period was 15:41.

The expression levels of *Hd3a* and *RFT1* were quantified in plants grown under field conditions in Milan, between May and July, when flowering is induced. Expression of *Hd3a* and *RFT1* rapidly decreased in Nipponbare as day length became longer (Fig. 2). Conversely, most Italian varieties showed induction of *Hd3a* and *RFT1* expression, and peak expression was observed 96 days after sowing (DAS) in most of them. Varieties bearing wild type alleles of *Hd1*, *Ghd7* and *Ghd8* had higher levels of *Hd3a* and *RFT1* mRNA compared to Nipponbare, indicating that de-repression of florigens is not exclusively caused by mutations in these regulators (Fig. 2B, C).

Positive correlations between the amount of *Hd3a* and *RFT1* mRNA and heading dates of the mini-panel grown under NLD were observed, and the highest correlations were determined for *RFT1* at 68 DAS (Table 2). These data indicate that *Hd3a* and *RFT1* are not repressed in varieties of the mini-panel and expression of both genes likely contributes to flowering under NLD.

**Table 2.** Correlation between *Hd3a* and *RFT1* gene expression levels and heading date

	NLD						SD	
	40DAS	54DAS	68DAS	82DAS	96DAS	110DAS	33DAS	47DAS
r^2^ *Hd3a*/heading	0.0003	0.0493	0.2879	0.1871	0.2862	0.1472	0.0144	*0.6134*
r^2^ *RFT1*/heading	0.0031	0.1973	*0.5686*	*0.3838*	0.2757	0.0167	*0.4310*	*0.7158*

NLD: natural long days; SD: short days; DAS: days after sowing. Highest correlations are indicated in italic.

**Fig. 3.**
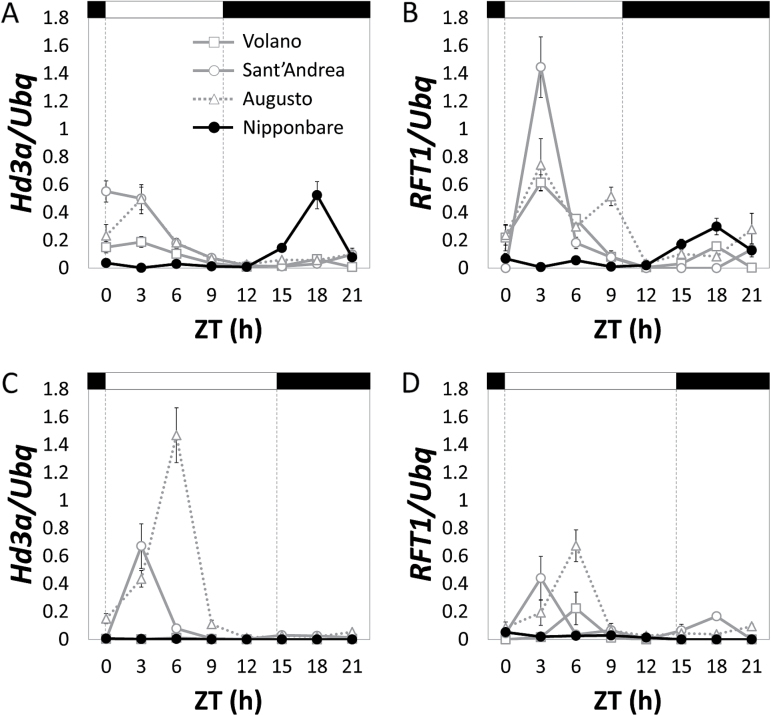
Diurnal patterns of *Hd3a* and *RFT1* mRNA expression in Italian varieties under SD and LD. (A) Diurnal quantification of *Hd3a* mRNA expression under SD. (B) Diurnal quantification of *RFT1* mRNA expression under SD. (C) Diurnal quantification of *Hd3a* mRNA expression under LD. (D) Diurnal quantification of *RFT1* mRNA expression under LD. Quantification of expression levels was determined in Volano, Sant’Andrea and Augusto compared to Nipponbare. Bars on top indicate day (white) or night (black). ZT indicates hours from dawn (*Zeitgeber*). *Ubiquitin* (*Ubq*) was used to normalize gene expression levels.

It has been previously demonstrated that under SD conditions *RFT1* expression was lower compared to *Hd3a* in wild type plants. Further reduction of its expression by RNAi caused no delay of flowering, indicating that *RFT1* is not contributing to flowering promotion under SD (Komiya *et al.*, 2008). In Italian varieties, the correlation between heading dates and *RFT1* expression was higher than with *Hd3a* expression at two time points under continuous SD (Table 2). Expression of upstream regulators of the florigens was poorly correlated to heading dates (Supplementary Table S4). Therefore, the diurnal mRNA expression profiles of *Hd3a* and *RFT1* were determined under both LD and SD. In addition to Nipponbare, which shows a high PS index, three Italian varieties with a low PS index were selected for this experiment, regardless of genotype. *Hd3a* was induced in all varieties to levels similar to those observed in Nipponbare (Fig. 3A). Additionally, high levels of *RFT1* expression in the three Italian varieties (Fig. 3B) were observed, indicating that *RFT1* transcription was strongly de-repressed under SD and exceeded that of Nipponbare. Plants grown under LD showed induction of *Hd3a* and *RFT1* transcription (Fig. 3C, D). The overall expression levels of *RFT1* were higher under SD than under LD (Fig. 3B, D). In Italian varieties, de-repression of *Hd3a* and *RFT1* transcription occurs under both long and short photoperiods in the morning, differently from the normal day length-induced peak in the evening in Nipponbare. Induction of *RFT1* expression is not only a consequence of activation by the LD pathway but rather the result of its transcriptional de-repression in the background of varieties adapted to northern latitudes.

### *Hd1* represses *Ehd1* under natural long days

Several varieties could flower under NLD despite bearing wild type alleles of all LD floral repressor genes analysed, indicating the existence of additional factors that contribute to reduce the sensitivity to photoperiod (Fig. 2). Whether these factors could act through upstream transcriptional regulators of *Hd3a* and *RFT1* was tested by quantifying the expression levels of *Ehd1* and *Hd1*. Expression of *Ehd1* was transiently induced, similarly to *Hd3a* and *RFT1* (Fig. 2A), and in many varieties its expression was high regardless of the presence of *Ghd7* and/or *Ghd8* wild type alleles (Fig. 2A, E, H). Quantification of *Hd1* in the same varieties showed that its expression was strongly reduced and often completely abolished compared to Nipponbare (Fig. 2D). Expression of other LD repressors was not reduced (Fig. S2), supporting the idea that additional factors influence flowering and PS by repressing *Hd1* expression and by promoting *Ehd1* expression.

**Fig. 4.**
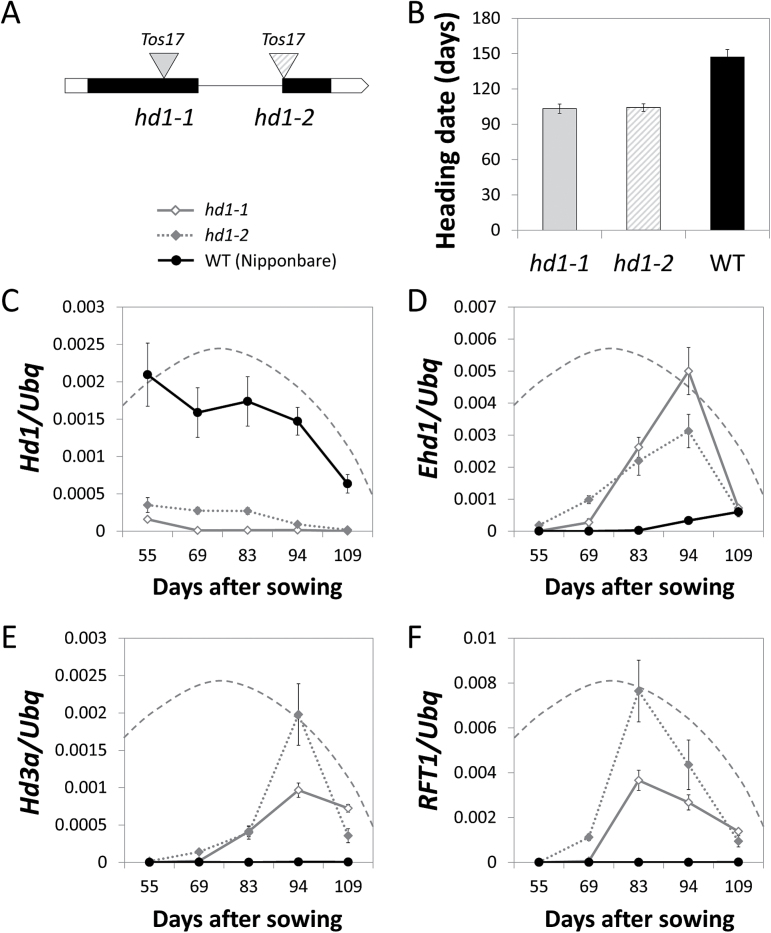
*Hd1* represses *Ehd1* under long days in the field. (A) Diagram of the *Hd1* locus indicating the position of *Tos17* retrotransposons in *hd1-1* and *hd1-2* alleles. Black and white boxes indicate exons and UTRs, respectively. (B) Heading dates of *hd1-1* and *hd1-2* mutants grown under NLD and compared to Nipponbare (wt). Quantification of *Hd1* (C), *Ehd1* (D), *Hd3a* (E) and *RFT1* (F) mRNA levels in *hd1-1*, *hd1-2* and Nipponbare in seasonal time courses. Samples were collected at the indicated days after sowing, two hours after dawn. Dashed lines indicate the dynamics of day length during the sampling period.

Whether increased expression of *Ehd1* mRNA was caused by reduced expression of *Hd1* was tested using *hd1* mutant alleles isolated from Nipponbare. Two loss-of-function mutants in which *Tos17* retrotransposons were inserted in the first and second exon, respectively, were grown under natural long days in Milan (Fig. 4A). Both mutant alleles showed accelerated heading compared to Nipponbare (Fig. 4B) and transiently expressed *Hd3a* and *RFT1* in leaves (Fig. 4E, F). The expression levels of *Ehd1* were also higher compared to those of wild type plants (Fig. 4D), indicating that *Hd1* repressed *Ehd1* under NLD and that such repression was sufficient to prevent flowering at higher latitudes.

### Single mutations of LD repressor genes are sufficient to promote flowering at higher latitudes

**Fig. 5.**
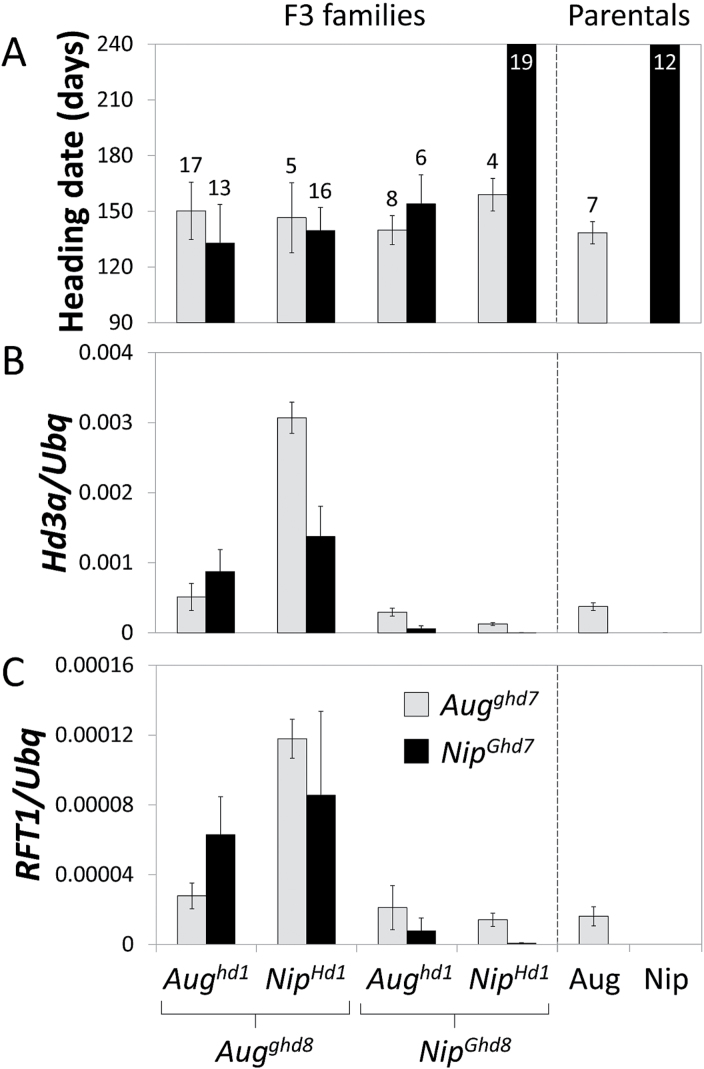
Single mutations in *Hd1*, *Ghd7* or *Ghd8* are sufficient to accelerate flowering under long days. (A) Heading dates of F3 plants obtained from a cross between Augusto (Aug) and Nipponbare (Nip) grown under LD. Numbers on top of the columns indicate the number of plants scored. Quantification of *Hd3a* (B) and *RFT1* (C) mRNA expression in the same plants as in (A). F3 plants bearing functional alleles for the three repressors and Nipponbare plants had not flowered after 240 days. Samples were collected 66 days after sowing and *Ubiquitin* (*Ubq*) was used to normalize gene expression levels.

Combinations of *hd1*, *ghd7* and *ghd8* mutations seemed to contribute to higher expression of *Hd3a* under NLD, as shown in the group of varieties bearing several non-functional alleles (Fig. 2I) compared to those bearing a single non-functional allele (Fig. 2F) or only functional alleles (Fig. 2B). Nipponbare (*Hd1*, *Ghd7*, *Ghd8*) and Augusto (*hd1*, *ghd7*, *ghd8*) showed different heading dates under SD, LD and NLD, opposing patterns of *Hd3a* and *RFT1* expression under NLD and very different PS (Fig. 1 and Fig. 2I, J). To assess if pyramiding of *hd1*, *ghd7* and *ghd8* mutations is necessary to promote flowering under LD, Augusto and Nipponbare were crossed, F2 individuals bearing all combinations of *hd1*, *ghd7* and *ghd8* mutations were selected and heading dates of F3 families grown under constant LD were scored. Plants bearing only functional LD repressor genes recapitulated the late flowering phenotype of Nipponbare and did not express *Hd3a* or *RFT1*, whereas plants bearing at least one mutant repressor flowered earlier (Fig. 5A) and expressed *Hd3a* and *RFT1* at higher levels (Fig. 5B, C). The *Hd6* mutant allele from Nipponbare segregated in F3 families, but it did not affect heading dates or gene expression levels (data not shown). The homozygous mutant *ghd8* from Augusto had the strongest effect to increase *Hd3a* and *RFT1* expression, either in combination with *ghd7* or alone (Fig. 5B, C). Taken together, these data suggest that single mutations in *Hd1*, *Ghd7* or *Ghd8* or reduction of *Hd1* expression are sufficient to de-repress *Hd3a* and *RFT1* expression and to promote flowering at higher latitudes.

### Loss-of-function alleles of *Ghd8*, *Ghd7* and *PRR37* but not of *Hd1* are associated with early heading at northernmost sites in Europe

**Fig. 6.**
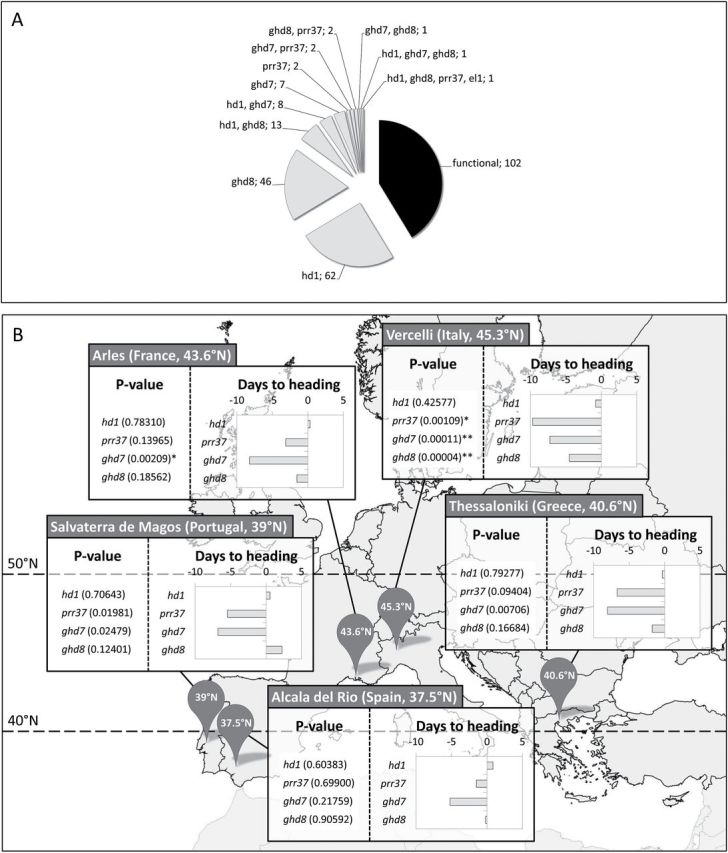
Targeted association mapping in a European rice collection. (A) Frequency of genotypes determined at floral repressor loci in a European rice collection comprising 247 accessions. (B) p-values of association between genotypes and time to flowering determined at five locations in Europe. Asterisks indicate significant associations (* indicates p≤0.005, ** indicate p≤0.0005). Graphs on the right within each box indicate the mean effect of the mutant allele on days to heading as determined by allele substitution tests.

To assess how long day repressors impact on heading dates across a latitudinal cline at higher latitudes, a germplasm collection comprising 247 varieties cultivated in Europe was used (Ahmadi *et al.*, 2011; http://eurigendb.cirad.fr). The frequency of *hd1*, *prr37*, *ghd7*, *ghd8* and e*l1* mutant alleles was determined for all varieties (Supplementary Tables S1 and S2). Of these, 145 (58.7%) carried at least one non-functional allele (Fig. 6A), a result very similar to that observed using the mini-panel. Non-functional alleles of *Hd1* were the most frequent followed by *ghd8*, *ghd7*, *prr37* and *el1* (Fig. 6A). Extensive pyramiding of loss-of-function mutant alleles was observed, including a variety (Timich 108 from Romania) bearing four mutations (*hd1*, *ghd8*, *prr37*, *el1*; Fig. 6A, Supplementary Table S1).

Selected varieties of the European collection were grown across a latitudinal cline at five locations in Europe, comprising Vercelli (Italy, 45.3°N), Arles (France, 43.6°N), Thessaloniki (Greece, 40.6°N), Salvaterra de Magos (Portugal, 39°N) and Alcala del Rio (Spain, 37.5°N). Flowering time data were recorded and made publically available at http://tropgenedb.cirad.fr and in Supplementary Table S1 (Feyt *et al.*, 2001). For each gene, all loss-of-function alleles were grouped into a single NF and genotypic data were used for targeted association analysis. Results of association statistics are presented in Fig. 6B, following a MLM2. The p-values of association calculated according to other models are reported in Supplementary Table S3. The mean effect of allelic substitutions between wild type and mutant alleles on days to heading were calculated as the phenotypic difference between the two homozygotes, by setting to 0 the effect of the functional allele and computing the effect of the non-functional allele. Depending on the location, different alleles were significantly associated with early heading (Fig. 6B). At the northernmost location, strong associations were detected for *ghd8*, *ghd7* and *prr37*. Only *ghd7* mutations were associated to early heading in Arles. No association was detected between *hd1* loss-of-function alleles and early heading at any location. A latitudinal gradient of significance was observed when considering the p-values of association for *ghd7* and *ghd8* (Supplementary Table S3). Therefore, loss-of-function alleles of flowering time genes are differentially affecting cycle duration, depending on the latitude and mutant alleles of *ghd7* and *ghd8* are the most effective to accelerate flowering at the northern limits of rice cultivation in Europe, but their effect quickly decreases with latitude.

## Discussion

Rice is largely cultivated in Europe and has a major impact on the economy of several regions. Its adaptation to higher latitudes has required the targeted manipulation of several genes. This study provides a comprehensive characterization of the natural genetic variation of loci encoding LD floral repressor genes and shows how selection of non-functional alleles in European germplasm could be part of the mechanism that allowed rice cultivation at northern sites in Europe. These data can be the basis to design varieties better adapted to such environments. Additionally, the study of *Hd1* alleles showing reduced or null expression has allowed for better definition of a regulatory node in the LD pathway, suggesting that *Hd1* can repress *Ehd1* to reduce expression of the florigens under LD.

### Genetic variation of *Hd3a* and *RFT1* expression contributes to identification of the existence of novel factors regulating flowering

Flowering of rice is promoted upon expression of *Hd3a* and *RFT1*. These genes are physically located very close to each other and are likely the product of chromosomal duplication. Yet, their function has diversified during evolution and mutant analysis has helped elucidate the role of each gene under different photoperiods. Plants in which *Hd3a* expression is reduced by RNAi delay flowering under SD, but flower normally under LD. Conversely, reduction of *RFT1* mRNA expression delays flowering under LD, but not under SD (Komiya *et al*., 2008, 2009). Transgenic plants in which both genes are down-regulated produce a strong additive effect and do not flower, clearly indicating that they have a fundamental role for flowering in rice (Komiya *et al.*, 2009). These results account for the little functional genetic variation that has been detected at loci encoding *Hd3a* and *RFT1* in cultivated varieties (Takahashi *et al.*, 2009; Ogiso-Tanaka *et al.*, 2013; Naranjo *et al.*, 2014). Still, genetic diversity of *RFT1* is higher than that of *Hd3a* and functional polymorphisms at the *RFT1* locus have been recently identified that disrupt protein function and lead to delayed flowering under LD (Hagiwara *et al.*, 2009; Ogiso-Tanaka *et al.*, 2013). Also several species from the genus *Oryza* bear mutations in *RFT1* that lead to non-functional proteins (Ogiso-Tanaka *et al.*, 2013). These data indicate that *RFT1* might not be required under specific environmental conditions, particularly at tropical latitudes where *Hd3a* is the major factor promoting flowering.

For these reasons, genetic variation at *Hd3a* or *RFT1* loci was not assessed in this study, but given the importance of their expression levels to regulate floral induction, detailed expression analyses were performed and expression curves were designed for several varieties. The results indicate that varieties adapted to grow in Europe induce both *Hd3a* and *RFT1* under NLD and such induction correlates with flowering. However, variation in expression patterns could not be exclusively correlated to genotypes at known repressor loci, indicating that the contribution of other regulators, likely including transcriptional activators or as yet unknown genes, has to be taken into consideration.

Genetic evidence indicated that reducing *Hd1* expression is sufficient to adapt rice flowering to higher latitudes because *Hd1* could repress *Ehd1* under NLD and consequently, *Hd3a* and *RFT1*. This finding highlights the central role of *Ehd1* in the LD regulatory network, where repression from *Ghd7*, *Ghd8* and *Hd1* converge. It further suggests the existence of *Hd1* repressors that might be useful tools to breed varieties adapted to long photoperiods, bypassing the need to introduce mutations in *Hd1* or other repressors. European varieties could be useful genetic tools to identify such novel regulators.

Seasonal induction of *Hd3a* and *RFT1* was transient in all varieties tested. Induction started between 54 and 68 DAS, reached a maximum 96 DAS in most varieties and then rapidly decreased. This pattern seems to be common to other varieties and locations (Ogiso-Tanaka *et al.*, 2013), and suggests that maintenance of high levels of *Hd3a* and *RFT1* expression in leaves is not required to sustain the flowering process. Upon commitment of shoot apical meristems to reproductive development, *Hd3a* and *RFT1* expression is likely not necessary any longer. Transcriptional dynamics were largely shared by both *Hd3a* and *RFT1*, suggesting a common mechanism for their temporary transcriptional induction in varieties adapted to LD. The molecular nature of this system is unknown but one possibility is that an epigenetic mechanism might temporarily allow access of *RFT1* and *Hd3a* chromatin to transcriptional activators. Given the close proximity of these two genes, such mechanism might be operating on both of them.

Under NLD, *Hd3a* and *RFT1* expression quickly dropped in Nipponbare, a variety strongly sensitive to day length, but under developmental or diurnal time courses under SD their levels were high and promoted flowering. Correlation of heading dates with *RFT1* expression levels was higher than with *Hd3a* and peak levels of *RFT1* transcription in several Italian varieties were higher than those of Nipponbare (peak expression of *Hd3a* and *RFT1* showed a shift of up to six hours compared to Nipponbare, a behaviour that we cannot currently explain but that could be related to defects in diurnal time measurement). These results indicate that *RFT1* can be strongly activated also under SD conditions, depending on the genetic background and demonstrate that regulation of its transcription can be very adaptable.

### Mutations in *Ghd7*, *Ghd8* and *PRR37* are strongly associated to early heading varieties at northern latitudes but not at southern latitudes in Europe

Association mapping between time to flowering and targeted loci across a latitudinal cline in Europe allowed assigning a relative weight to LD repressors. *Ghd8*, *Ghd7* and *PRR37* were detected as major repressors of flowering at the northernmost limits of rice cultivation in Europe, but not at southern locations. These genes have been useful tools for breeders to reduce cycle length and varieties bearing the mutant alleles could spread closer to the northern limits of rice cultivation in Asia (Fujino and Sekiguchi, 2008; Xue *et al.*, 2008; Wei *et al.*, 2010; Ebana *et al.*, 2011; Fujino *et al.*, 2013; Gao *et al.*, 2014). However, whereas strong associations between genotypes and heading were detected for *Ghd7*, *Ghd8* and *PRR37* at the northernmost location in Europe, the effect of *ghd8* and *prr37* was milder in France, where only *ghd7* was statistically associated to fast-cycling accessions. At even southern locations, including Greece, Portugal and Spain, no statistically significant association could be detected. These data suggest that the photoperiodic network can finely tune flowering responses and has the capacity to detect small changes in day length. Since seasonal variation of photoperiod is different depending on latitude, it is likely sufficient to modulate flowering to the extent that loss of function alleles that are crucial at ~45°N to promote flowering become less relevant at 40°N or lower latitudes. It is however important to note that statistical thresholds do not abolish the biological value of some alleles. For example, despite not being statistically significant according to the models used in this study, *ghd7* mutant alleles should be considered useful tools to accelerate flowering in Greece.

High natural genetic variation is associated to the *Hd1* locus and contributed to diversification of flowering time in many varieties, including those adapted to northern regions (Yano *et al.*, 2000; Fujino *et al.*, 2008; Takahashi *et al.*, 2009; Fujino *et al.*, 2010; Zhao *et al.*, 2011; Naranjo *et al.*, 2014). *Hd1* represses flowering under LD, and mutant alleles confer an advantage to varieties adapted to short-growing seasons. Several European varieties bear mutant alleles of *hd1* and genetic evidence also showed that such mutations can increase *Ehd1* transcription and promote flowering. However, targeted association mapping did not detect any significant association between early heading and mutant alleles at this locus. This is likely the result of population structure of the European collection used in this study (Courtois *et al.*, 2012). European varieties are mostly *temperate japonica* accessions that have been further distinguished into four sub-groups, American, European 1 and 2, and Admixed, based on SSR and SNP markers (Courtois *et al.*, 2012). The American group comprises non-European accessions that contributed *hd1* mutant alleles to Europe. Accessions from this group, which have tropical ancestors in their background, are also consistently later than accessions from the other groups (Courtois *et al.*, 2012). When associations with *hd1* mutants were corrected for population structure and kinship, they were not anymore significant. Therefore, the full statistical significance and impact of *Hd1* alleles in Europe remains to be determined. This specific case highlights some limitations of association analysis, when population structure is strongly correlated with phenotypic differences.

### Mutant selection as a tool to expand the cultivated area of rice

Field data and analysis of F3 families indicated that single mutations in *hd1*, *ghd7* or *ghd8* are sufficient to accelerate flowering under NLD or LD, even if not sufficient to produce very fast cycling accessions. Complex genotypes where up to four repressors were mutated in a single variety were observed when mining European germplasm. Yet, the significance of pyramiding of floral repressors remains to be determined. Varieties bearing combinations of *ppr37* and *ghd7* mutant alleles have been reported as very early flowering and photoperiod insensitive, possibly indicating that targeting these regulators is sufficient to generate genotypes suitable for cultivation at very high latitudes (Ebana *et al.*, 2011; Kim *et al.*, 2013; Koo *et al.*, 2013). Consistently, among European varieties, Auzgusta and Russo1 (*ghd7 prr37*) were among the earliest flowering accessions when grown in Vercelli. However, *ghd7* and *prr37* mutant alleles seem to be underrepresented among European rice varieties, at least compared to *hd1* and *ghd8* mutant alleles. Increasing their frequency, and particularly that of *prr37*, might facilitate reduction of cycle duration or allow moving cultivation of specific varieties to even northern latitudes.

## Supplementary Material

Supplementary data can be found at *JXB* online.

Supplementary Fig. S1. Natural genetic variation of repressor genes belonging to the mini-panel compared to Nipponbare.

Supplementary Fig. S2. Expression dynamics of *Ghd7* (A) and *PRR37* (B) in Italian varieties grown under NLD. All varieties bear functional alleles of floral repressor genes.

Supplementary Table S1. Allelic variants of *Hd1*, *PRR37*, *Ghd7*, *Ghd8* and *EL1* and heading dates of 247 European rice varieties grown at 5 locations in Europe.

Supplementary Table S2. Strategies and primers used for genotyping mutant alleles of LD repressor genes.

Supplementary Table S3. p-values obtained from targeted association mapping analyses using different statistical models. NF: non-functional; GLM: General Linear Model, MLM: Mixed Linear Model.

Supplementary Table S4. Correlation between *Ehd1* and *OsMADS51* gene expression levels and heading dates of Italian varieties grown under continuous SD.

Supplementary Table S5. Primers used for quantification of mRNA expression.

Supplementary Data
