# Supplementary material for: Loss of floral repressor function adapts rice to higher latitudes in Europe
Source: J Exp Bot. 2015 Mar 1;66(7):2027–39. doi: 10.1093/jxb/erv004 (PMC4378634; doi:10.1093/jxb/erv004)
Supplement: Supplementary Data [file supp_66_7_2027__index.html]

Loss of floral repressor function adapts rice to higher latitudes in Europe — Supplementary Data 

# Loss of floral repressor function adapts rice to higher latitudes in Europe

## Supplementary Data

Data files

**Files in this Data Supplement:**

- Supplementary Data - Supplementary Data
- Supplementary Data - Supplementary Data
