# Supplementary material for: Loss of floral repressor function adapts rice to higher latitudes in Europe
Source: J Exp Bot. 2015 Mar 1;66(7):2027–39. doi: 10.1093/jxb/erv004 (PMC4378634; doi:10.1093/jxb/erv004)
Supplement: Supplementary Data [file supp_erv004_erv004_SUPPLEMENTARY_FIGURES_with_legends.pdf]

Hd1

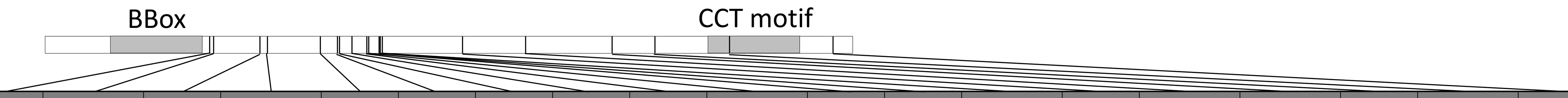

Position	242	248	316	327	404	430	433	451	473	476	492	493	496	614	706	834	897	1006	1159	
aa sub.	C81Y	R83Q	H106Y	-	D135G	L144I	L145I	D151N	N158S	-	-	N165D	D166N	R205Q	F.S.	F.S.	R299S	-	G387S	No. of cvs.
Hd1-Nip	G	GC	C	-	A	C	C	G	A	-	T	A	GAC	G	-	3T	A	3AAG	G	0
Hd1-I	G	GC	T	36bp	A	C	C	G	A	-	T	A	GAC	G	-	3T	A	3AAG	G	1
Hd1-II	G	GC	T	36bp	A	C	C	G	A	-	T	A	GAC	G	43bp	3T	A	3AAG	G	2
Hd1-III	G	AA	C	36bp	G	A	A	G	A	123bp	C	G	AAT	G	-	3T	C	2AAG	A	1
Hd1-IV	G	AA	C	36bp	G	A	A	A	A	123bp	C	G	AAT	A	-	3T	C	2AAG	A	3
Hd1-V	G	AA	C	36bp	G	A	A	G	A	123bp	C	G	AAT	G	-	1T	C	3AAG	A	2
Hd1-VI	G	AA	C	36bp	G	A	A	A	A	123bp	C	G	AAT	A	-	1T	C	3AAG	A	4
Hd1-VII	A	AA	C	36bp	G	A	A	A	G	114bp	C	G	AAT	A	-	3T	C	3AAG	A	3

PRR37

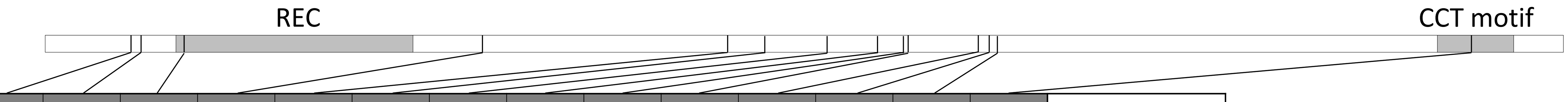

Position	125	140	204	641	1002	1055	1147	1221	1259	1266	1369	1385	1397	2094	
aa sub.	I42T	R47P	D68E	N214S	-	A352V	A383T	-	G420D	-	M457V	L462P	S466I	-	No. of cvs.
PRR37-Nip	T	G	T	A	A	C	G	T	G	C	A	T	G	C	1
PRR37-I	T	C	T	A	A	C	G	T	G	C	A	T	G	C	10
PRR37-II	C	C	G	G	A	C	A	T	A	T	G	C	T	C	4
PRR37-III	C	C	G	G	G	T	A	A	G	T	G	C	G	T	1

Ghd7

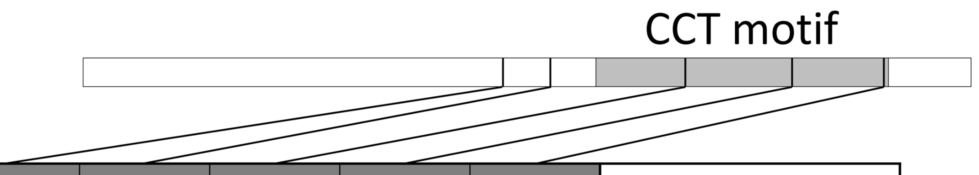

Position	365	406	521	618	697	
aa sub.	E122G	G136S	D174V	Y206@	P233A	No. of cvs.
Ghd7-Nip	A	G	A	C	C	12
GHd7-0	-	-	-	-	-	2
Ghd7-I	A	G	A	A	C	1
Ghd7-II	G	A	T	C	G	1

COL4

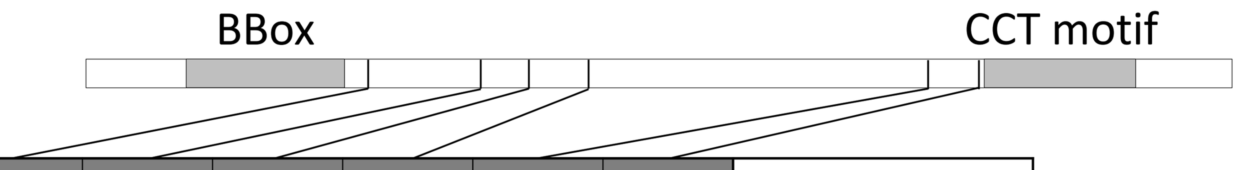

Position	243	344	385	438	733	777	
aa sub.	-	A115V	P129T	-	S245A	K259N	No. of cvs.
COL4-Nip	C	C	C	T	T	G	10
COL4-I	A	C	C	C	G	G	2
COL4-II	C	T	A	C	G	C	4

Ghd8

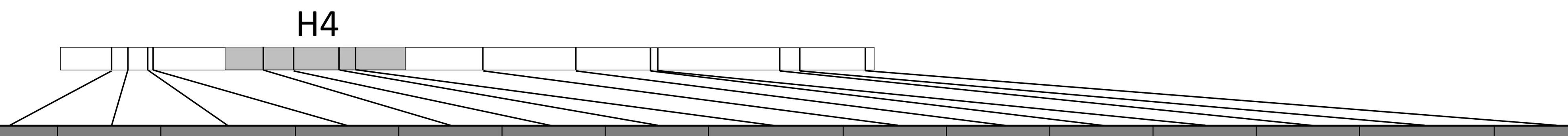

Position	56	74	93	96	222	256	306	323	461	566	648	656	789	811	884	
aa sub.	L19S	E25A	F.S.	-	-	S86A	-	F.S.	D154A	Y189S	-	D219A	-	-	N295S	No. of cvs.
Ghd8-Nip	T	A	-	T	G	T	A	-	A	A	C	A	C	6GGC	A	10
Ghd8-I	T	A	-	T	T	T	A	-	A	A	C	A	A	6GGC	A	1
Ghd8-II	T	A	19bp	T	G	T	A	-	A	A	C	A	C	6GGC	A	4
Ghd8-III	C	C	-	G	G	G	C	1bp	C	C	T	C	C	3GGC	G	1

Supplementary Fig. S1. Natural genetic variation of repressor genes belonging to the mini-panel compared to Nipponbare.
The gene structure of each repressor is indicated on top of the corresponding table. Numbers indicate the nucleotide position from the start codon. Blue boxes indicate synonymous SNPs; yellow boxes indicate non-synonymous SNPs; green boxes indicate insertions; orange boxes indicate deletions; red boxes indicate polymorphisms introducing premature stop codons or altering the reading frame and therefore creating non-functional alleles.

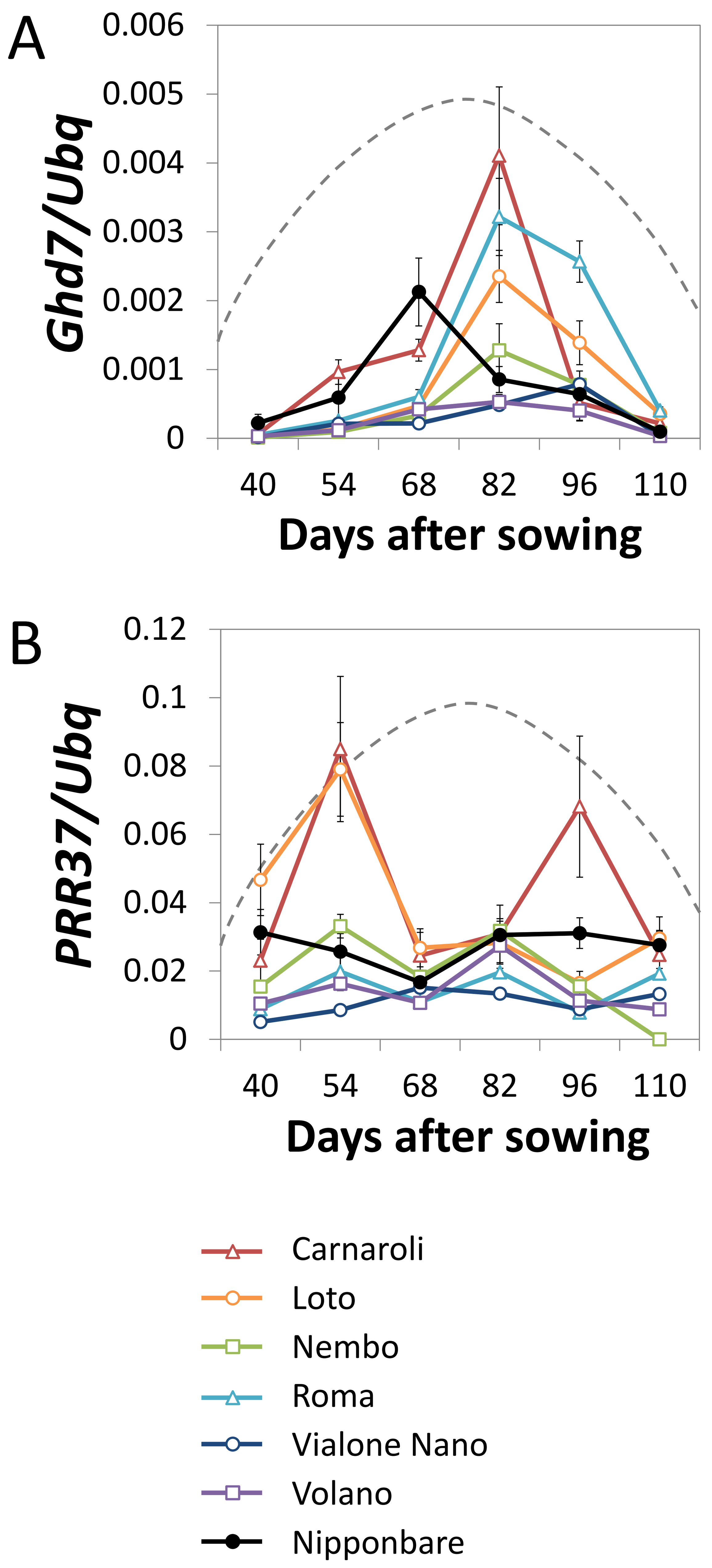

Supplementary Fig. S2. Expression dynamics of *Ghd7* (A) and *PRR37* (B) in Italian varieties grown under NLD. All varieties bear functional alleles of floral repressor genes.
